# Modularity of *Escherichia coli *sRNA regulation revealed by sRNA-target and protein network analysis

**DOI:** 10.1186/1471-2105-11-S7-S11

**Published:** 2010-10-15

**Authors:** Timothy H Wu, Ian Yi-Feng Chang, Li-chieh Julie Chu, Hsuan-Cheng Huang, Wailap Victor Ng

**Affiliations:** 1 Institute of Biomedical Informatics, National Yang Ming University, Taipei, Taiwan; 2 Department of Biotechnology and Laboratory Science in Medicine and Institute of Biotechnology in Medicine, National Yang Ming University, Taipei, Taiwan; 3 Center for Systems and Synthetic Biology, National Yang Ming University, Taipei, Taiwan

## Abstract

**Background:**

sRNAs, which belong to the non-coding RNA family and range from approximately 50 to 400 nucleotides, serve various important gene regulatory roles. Most are believed to be *trans*-regulating and function by being complementary to their target mRNAs in order to inhibiting translation by ribosome occlusion. Despite this understanding of their functionality, the global properties associated with regulation by sRNAs are not yet understood. Here we use topological analysis of sRNA targets in terms of protein-protein interaction and transcription-regulatory networks in *Escherichia coli *to shed light on the global correlation between sRNA regulation and cellular control networks.

**Results:**

The analysis of sRNA targets in terms of their networks showed that some specific network properties could be identified. In protein-protein interaction network, sRNA targets tend to occupy more central positions (higher closeness centrality, *p-val *= 0.022) and more cliquish (larger clustering coefficient, *p-val *= 0.037). The targets of the same sRNA tend to form a network module (shorter characteristic path length, *p-val *= 0.015; larger density, *p-val *= 0.019; higher in-degree ratio, *p-val *= 0.009). Using the transcription-regulatory network, sRNA targets tend to be under multiple regulation (higher indegree, *p-val *= 0.013) and the targets usually are important to the transfer of regulatory signals (higher betweenness, *p-val *= 0.012). As was found for the protein-protein interaction network, the targets that are regulated by the same sRNA also tend to be closely knit within the transcription-regulatory network (larger density, *p-val *= 0.036), and inward interactions between them are greater than the outward interactions (higher in-degree ratio, *p-val *= 0.023). However, after incorporating information on predicted sRNAs and down-stream targets, the results are not as clear-cut, but the overall network modularity is still evident.

**Conclusions:**

Our results indicate that sRNA targeting tends to show a clustering pattern that is similar to the human microRNA regulation associated with protein-protein interaction network that was observed in a previous study. Namely, the sRNA targets show close interaction and forms a closely knit network module for both the protein-protein interaction and the transcription-regulatory networks. Thus, targets of the same sRNA work in a concerted way toward a specific goal. In addition, in the transcription-regulatory network, sRNA targets act as "multiplexor", accepting regulatory control from multiple sources and acting accordingly. Our results indicate that sRNA targeting shows different properties when compared to the proteins that form cellular networks.

## Background

A subclass of non-coding RNAs, small non-coding RNAs (sRNAs), has been found to play important regulatory roles in gene expression in prokaryotes. Up to the present, sRNAs, which range in size from approximately 50 to 400 nucleotides, have been detected and predicted in both archaea and bacteria [1,2]. Most studies of regulatory sRNAs have been carried out in *E. coli *and approximately 80 species of sRNAs have been identified in this organism. They function as either positive or negative regulators of proteins synthesis or mRNA stability (Reviewed in [3]). However, most are believed to be *trans*-regulating and execute their function by complementing their target mRNAs in order to prevent the loading of ribosomes and thereby inhibiting translation. sRNAs have a number of crucial roles such as regulation of iron homeostasis, control of outer membrane protein biogenesis, regulation of sugar metabolism, quorum sensing, and control of survival in stationary phase [4].

The properties of sRNA regulation can be studied globally at the cellular level, where certain topological features may be discovered. In this approach, proteins are modeled as nodes and their interactions act as an edge, which sheds light on whether some of the biological properties may be correlated with the network topology. For example, Jeong *et al. *noted that lethality in yeast, which was induced by removal of a protein, positively correlates with its connectivity in the protein-protein interaction network (PPIN) [5]. In this context, in *C. elegans*, the "hub" genes, from an evolutionary perspective, are more conserved when compared with their orthologues [6]. Studies of miRNA targets in the context of protein-protein interaction networks have led to the discovery that miRNAs tend to regulate intra-modular hubs in the network, and that interacting proteins tend to be regulated by similar miRNA regulation systems [7]. Using a similar approach in this study, we explored the topological properties of sRNA targets in terms of the protein-protein interaction network and the transcription regulatory network in order to reveal whether sRNAs in *E. coli *possess similar properties.

## Methods

### Material preparation

A reliable set of sRNA targets were obtained then mapped onto the protein-protein interaction network and transcription regulatory networks. Out of a total of 79 sRNAs listed for *E. coli K12 MG1655*, there are sixty-five experimentally validated sRNA-sRNA target pairs (consisting of 57 unique target genes) and these were obtained from the sRNAMap database [8]. To enlarge our dataset of sRNA targets, sRNA-sRNA target pairs were predicted using the *TargetRNA *program [9]. The 616 predicted sRNA-target pairs (532 unique genes) were further analyzed by *IntaRNA *[10] to evaluate the authenticity of the putative targets (*p *value < 0.05 obtained based on a simulation of random targets). A total of 240 sRNA-target pairs were obtained, which included the 65 experimentally validated pairs as mentioned above. Since sRNA-mRNA complementarity may affect downstream gene translation, the target list was also expanded to include an "extended set" obtained by examination of downstream genes present in operons containing the sRNA targets. Finally, the sRNA targets were mapped to the protein-protein interaction data from *DIP *[11] and transcription regulatory interaction data from RegulonDB [12].

### Network topology measurements

To characterize the properties of sRNA targets among the networks, a set of calculations that evaluated the degree, closeness, betweenness, clustering coefficient, characteristic path length, density, and in-degree ratio were performed. The first four measurements are applied to single sRNA targets one at a time. Given a sRNA target, degree is the number of neighboring nodes to the target. This signifies how much influence this node can exert on or accept from other nodes. Closeness, which measures how "centered" is the node from all other nodes, is the reciprocal of average distance from the target node to all other nodes in the network. Betweenness is the number of shortest paths among all other nodes in the network that goes through the target. This represents the "message passing" that may goes through this sRNA target. Clustering coefficient is the ratio of the number of existing edges of the neighboring nodes to the total number of possible edges among them and a higher value suggests that the neighboring nodes form a network module. Other calculations demonstrate the properties of the subnets and are applied each time to all targets linked to a single sRNA. Characteristic path length is the average length of the shortest path to the targets. A shorter distance means that the sRNA regulates genes working in a concerted effort. Density is the ratio of number of edges among targets to all possible edges. This measures the level of connectivity of the targets and their sRNA. The in-degree ratio is the ratio of direct connections within the subnet to the outbound link to outside nodes. Statistical significance was evaluated through simulation by randomly selecting the same number of proteins as the real targets from the *E. coli *genome and applying the same set of calculations to these random targets to see where the true result lies in the distribution of these random results. A thousand simulations were done to obtain the Z-scores and *p-val*ues down to three significant figures.

Slightly different methods to the above calculations are applied to network where there are directional edges, in our case, the transcription regulatory network. The general changes to the definition of the measurements mentioned above are that only the outbound direction from a node is taken into consideration and the number of potentially linkable interaction (edges) is doubled. The notable difference is that the degree is divided into outbound degree and inbound degree and the clustering coefficient and characteristic path length are undefined. Statistical significance was evaluated similarly. Together, these centrality calculations reveal the topological features of the sRNA targets for both the protein-protein interaction network and the transcription-regulatory network in relation to other proteins in the networks.

## Results and discussion

### Properties of the sRNA targets in the networks

The analysis of experimentally confirmed sRNA targets in the protein-protein interaction network (PPIN) identified that some network properties, such as the modular distribution of sRNA and targets, emerged while others did not (Table 1). There is no evidence supporting sRNA targets being the hubs of the network (sRNAs do not have a higher degree). Neither is there evidence demonstrating sRNA targets being the center of communication (not significant for betweenness). However, the targets of the same sRNAs do exhibit some properties in that they seem to work as a module. One of these properties is that the distances among the targets of the same sRNA tend to be close since the average characteristic path length is 2.7 compared to 3.7 when picking nodes at random (*p-val *= 0.015). The density and in-degree ratio are also significant. Targets of the same sRNA are more closely linked than random nodes, thus they exhibit a higher density (*p-val *= 0.019), and also when compared to links outside of the targets, they are more highly connected (a higher in-degree ratio, *p-val *= 0.0090). This demonstrates that targets regulated by the same sRNA tend to work as a module in the protein-protein interaction network. sRNAs are likely to regulate genes central to cellular functioning together with other sRNA regulated genes from the protein interaction viewpoint.

**Table 1:** Centrality measurements for the RNA targets in the protein-protein interaction network

	All	Experimental			Experimental and predicted		
			
	mean	mean	**Sim**. mean	*p-val*	mean	**Sim**. mean	*p-val*
Deg	7.9	11	7.9	0.16	7.0	7.9	0.66
Bet	2.2e-03	4.2e-3	2.2e-3	0.098	2.2e-3	2.2e-3	0.44
Clo	0.28	**0.3**	**0.28**	**0.022**	0.27	0.28	0.59
CC	0.11	**0.21**	**0.11**	**0.037**	0.12	0.10	0.20
CPL	3.7	**2.6**	**3.7**	**0.015**	3.6	3.7	0.23
Den	6.5e-03	**0.14**	**6.5e-3**	**0.019**	**0.063**	**6.2e-3**	**0.028**
IDR	N/A	**6.5e-3**	**3.4e-4**	**9.0e-3**	**5.8e-3**	5.**0e-4**	**0.011**

I/T		28/65			85/240		

							

		**Experimental & extended**			**Exp & pred & both extended**		
			
**Deg**		**Mean**	**Sim**. **mean**	*****p-val*****	**Mean**	**Sim**. **mean**	***p-val***

Deg		9.3	7.9	0.28	7.4	7.9	0.62
Bet		3.4e-3	2.2e-3	0.14	2.0e-3	2.2e-3	0.57
Clo		0.29	0.28	0.051	0.27	0.28	0.87
CC		**0.19**	**0.11**	**0.027**	**0.14**	**0.10**	**0.017**
CPL		**3.0**	**3.7**	**0.020**	**3.4**	**3.7**	**5.0e-3**
Den		**0.085**	**7.0e-3**	**0.015**	**0.14**	**5.8e-3**	**0.0**
In-d		**4.0e-3**	**5.5e-4**	**0.032**	**9.5e-2**	**7.9e-4**	**0.0**

I/T		38/92			144/450		

Those with significant values (*p *< 0.05) are indicated in boldface and those close to a *p-value* of 0.05 are underlined. The extended targets are targets located downstream of experimental or predicted sRNA targets in the same operon. **Deg: Degree, Bet: Betweenness, Clo: Closeness, CC, Clustering coefficient, CPL: Characteristic path length Pat: Path length, Den: Density, IDR, In-degree ratio, I/T: sRNA in the network/total sRNA**

In the analysis of transcription regulatory network, several interesting properties were also observed for the experimentally confirmed sRNA targets (Table 2). When a slightly different set of calculations to those described above were applied, it came to us as a surprise that the inbound-degree, but not the outbound-degree, is significantly higher for sRNA targets than regular non-targets. In other word, sRNA targets are more likely subjected to regulations from transcription factors, and tend not to be at the beginning of regulatory signal propagation. Additionally, sRNA targets also rank higher for betweenness. This revealed that sRNA targets are often the "middle-man" of the regulatory signals with regard to the entire transcription regulatory network. The characteristic path length, unlike that of protein-protein interaction network, is undefined. However, targets regulated by the same sRNA also showed a tendency to work as a module, as can be seen by a higher density and higher in-degree ratio. Overall, sRNAs targets seem to be important "middle-men" that acts as "multiplexers", piping multiple regulatory signals into one regulatory control system. They also work (cross talk) with each other, which is also observable in the protein-protein interaction networks.

**Table 2:** Centrality measurements for the RNA targets in the transcription-regulatory interaction network

	All	Experimental			Experimental and predicted		
			
	Mean	Mean	**Sim**. Mean	*p-val*	Mean	**Sim**. mean	*p-val*
InD	2.3	**3.3**	**2.3**	**0.013**	**2.7**	**2.3**	**0.024**
OuD	2.3	5.8	2.6	0.13	2.9	2.5	0.32
Bet	6.6e-06	**7.1e-5**	**7.8e-6**	**0.012**	**2.7e-5**	**7.6e-6**	**0.038**
Clo	9.7e-02	8.2e-2	9.8e-2	0.55	8.4e-2	9.9e-2	0.66
Den	1.6e-03	**8.9e-3**	**1.6e-3**	**0.036**	2.3e-3	1.6e-3	0.13
IDR	N/A	**1.8e-3**	**1.9e-40**	**0.023**	6.3e-4	3.5e-4	0.15

I/T		42/65			110/240		

							

		**Experimental & extended**			**Exp & pred & both extended**		
			
		**Mean**	**Sim**. **mean**	***p-val***	**Mean**	**Sim**. **mean**	***p-val***

InD		**3.2**	**2.3**	**5.0e-3**	2.4	2.3	0.33
OuD		3.8	2.6	0.22	2.5	2.5	0.43
Bet		**4.5e-05**	**7.7e-06**	**0.040**	1.6e-05	7.7e-06	0.10
Clo		6.7e-02	9.9e-02	0.70	5.8e-02	9.9e-02	0.96
Den		6.1e-03	1.7e-03	0.071	**1.4e-02**	**1.5e-03**	**0.020**
IDR		**3.8e-03**	**3.5e-04**	**0.037**	**1.8e-02**	**5.6e-04**	**0.0**

I/T		66/92			251/450		

Those with significant values (*p *< 0.05) are indicated in boldface and those close to a *p-value* of 0.05 are underlined. The extended targets are targets located downstream of experimental or predicted sRNA targets in the same operons. **InD: Indegree, OuD: Outdegree, Bet: Betweenness, Clo: Closeness, Den, Density, IDR: In-degree Ratio**

When the calculations were applied beyond the experimentally validated targets, some measurements were similar to the results from the experimentally validated targets, while others were not (Table 1 and Table 2). On the surface, these predicted sRNA targets did not exhibit as many properties as the experimental ones (Additional file 1). A possible explanation is that the subnet properties require at least two targets in the network per sRNA to be eligible for calculation. There were many newly predicted sRNAs and targets not meeting this criterion. Furthermore, the predicted targets have to be connected to each other via network interactions. Many predicted sRNAs did not fit the above two criteria perhaps because of limitations in terms of prediction capability. It may require a larger amount of data than presently available for their properties to surface fully. Despite having a total of 45 targets eligible for subnet calculations in the transcription regulatory network, none of the targets interact with each other, hence their density and in-degree ratio are 0. When taking our extended set of targets into account, we approximately doubled the number of targets found on the protein-protein interaction network (57 to 106) and tripled that in the transcription regulatory network (68 to 185) (Additional file 1). More cases of subnet properties were then exposed as highly significant with a close exception being the characteristic path length for protein-protein interactions, which had a *p-value* of 0.084. For experimental targets with operon structures, namely ones with their downstream genes in both the protein-protein interaction and the transcription regulatory networks, the subnet properties are highly significant except for density in the transcription regulatory networks, which is slightly over the cutoff (*p-val *= 0.071). Additionally, clustering is highly significant in the protein-protein interaction network and indegree and betweenness are highly significant in the transcription regulatory network. Closeness in the protein-protein interaction network (*p-val *= 0.051) is also very close to the cutoff. The results also show the modularity of these sRNA targets in these networks. When all of the sRNA targets were pooled, including experimentally identified, predicted, and all of the extended operon downstream genes, the subnet properties are once again significant in both the protein-protein interaction network and the transcription regulatory network. These calculations reveal that the sRNA targets form a strong inter-connected module when the predicted targets and downstream genes were included.

### Robustness evaluation

To address the issue of data inaccuracies present in the network data, we conducted a sensitivity analysis to confirm the above observations. (Additional file 2) We randomly added and removed 5% and 10% of the edges in the protein-protein interaction and the transcription regulatory networks and applied the same calculations and statistical analysis. The results indicate that our conclusion is robust against inaccuracies in the datasets. The same conclusions were reached by similar robustness measurements for the predicted data (data not shown).

### OxyS targets in the protein-protein interaction network

**Figure 1:**
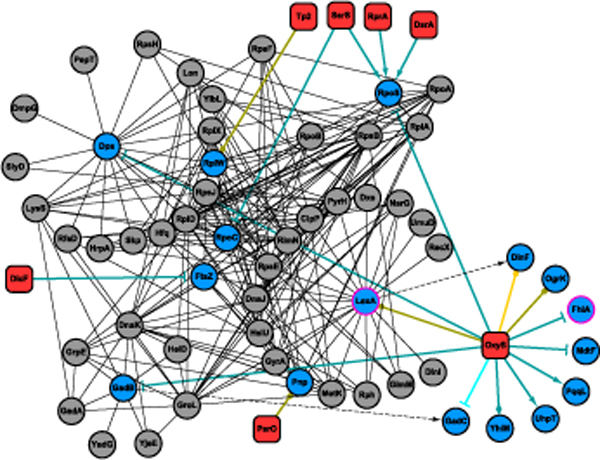
**sRNA *oxyS *and its target*s *in the gene-regulation and protein-protein interaction networks**. The dark green lines represent experimentally verified regulation and the dark yellow lines represent predicted regulation. The teal lines indicate indirect regulation (e.g., *dinF *downstream of *lexA *in the same operon). Yellow lines are also indirect regulation, but indicate genes of a predicted target. Dashed lines indicate regulated genes extended from an operon structure. Transcription factors under regulation of sRNA have pink borders. Arrow, T, and diamond heads represent positive, negative, and dual regulators, respectively. Circular heads represent predicted, thus unknown, regulation. OxyS regulation deals with multiple stress responses, such as oxidative and osmotic stresses.

To demonstrate our findings, we will discuss the concentrated interactions of a sRNA exemplar, OxyS, in the protein-protein interaction network. The interactions between the sRNA OxyS and its experimental and predicted targets, and neighbors of these targets are depicted in Figure 1 (The graph was generated with Cytoscape [13]). This network shows that OxyS is responsible for regulating a number of genes participating in the stress response. As an antioxidant defense pleiotropic regulator, OxyS is positively regulated by OxyR, which is a transcriptional activator under oxidative stress [14]. In the OxyS network, targets regulated by OxyS roughly forms three clusters with other interacting molecules. These clusters are centered on *rpoS*, *dps*, and *gadB*. Among these, dps is a DNA binding protein involved in a number of stress responses including oxidative stress [15] and fatty acid starvation [16]. GadB is the subunit of glutamate decarboxylase B, part of the glutamate-dependent acid resistance system 2, which protects the cell during anaerobic phosphate starvation. *RpoS *(σ^s^) encodes the RNA polymerase subunit sigma 38, which responses to osmotic and oxidative stresses. Since some of the genes participating in stress response, including *katG*, *dps*, *gadB *and *gorA*, are regulated by both σ^s ^and OxyR, it was suggested that repression of *rpoS *by OxyS may prevent redundant utilization of transcriptional regulators [14]. In addition, OxyR induces transcription of *fur*, whose product represses *rpoS *transcription [17,18]. Therefore, OxyR and OxyS together regulate *rpoS *on both the transcription level and the translation level. The gene *gadC*, which is downstream of *gadB *in the same operon, is required for decarboxylase-based acid resistance [19]. Other than the three major clusters in the interaction networks, several other targets not having protein interactions are also present. Two targets, *fhlA *and *rpoS*, encodes transcriptional regulators. FhlA is an activator required for the formate hydrogenlyase complex [20]. This metal-cofactor containing complex is primarily synthesized under anaerobic condition and may be detrimental to the cell during oxidative stress. Indirect repression by oxyS thus may reduce hydrogen-peroxide induced damage [21]. Three predicted targets, *lexA*, *ogrK*, and *dinF*, which are present in the network, are suggested to be regulated by *oxyS*. The genes *lexA *and *orgK *are predicted by *TargetRNA *and *IntaRNA*. *LexA *is part of the inducible DNA repair system. It is a global repressor of the SOS response regulon that allows bacteria to survive a sudden increase in DNA damage [22]. Upon DNA damage, such as that caused by UV light, the LexA repressor undergoes self-cleavage and the expression of SOS genes are thus activated [23]. *DinF *is downstream of *lexA *in term of genome position and is possibly a member of the family of MATE (multidrug and toxic compound extrusion) transporters induced by DNA damage [24,25]. It should also be noticed in the *oxyS *network that there are many other sRNAs that tend to work together as part of gene regulation. For instance, tp2 is predicted to regulate *rplW*, which encodes the 50 S ribosomal subunit protein L23. SsrS, RprA, and DsrA also regulate *rpoS *together with *oxyS*. In addition, SsrS also regulates *rpoC*, another subunit of RNA polymerase. Overall in the interaction network, we can see that *oxyS*, with other sRNAs, orchestrates a variety of genes participating in multiple stress responses, and these are mostly DNA damage associated. We can also see that targets represented in protein-protein interaction networks have many neighbors and their average clustering coefficient is approximately three times as high as average in the networks (3.5E-1 versus 1.1E-1). Other network properties were also found in the transcription regulatory network and an example is shown in Additional file 3.

## Conclusions

In this study, we measured the network properties of *E. coli *sRNA targets, both experimental and predicted, in terms of both the protein-protein interaction network and the transcription regulatory network. The data show that sRNAs in *E. coli *are likely to serve important regulatory roles in the cellular networks. Their targets appear to be positioned critically in the context of the protein-protein interaction and transcription regulatory networks. In protein-protein interaction network, sRNAs targets tend to be in close proximity (small characteristic path length), and tends to form a module (high density and in-degree ratio), which suggests functional specificity. The transcription regulatory network, aside from significant density and in-degree ratio like the protein-protein interaction network, also exhibits the interesting property that the inbound degree for the experimental targets and their downstream genes is significant. That is, it has a "multiplexor-like" role whereby it receives signals from multiple sources and act on their behalf. Summarizing for both networks, the prominent feature is the modularity of sRNA targets under regulation by the same sRNA. sRNA targets work in a cooperative manner and the targets of the same sRNA often interact with each other. Their neighbors also tend to cluster together.

There are some limitations inherent in this kind of study. For instance, the current method used the available static data with connections modeled as either connected or unconnected. The one or zero connectivity did not take into account temporal change or a spectrum of binding affinities. In addition, the sRNA target prediction methods might have some false positive predictions, resulting in evidence being "washed out" when the predicted sRNA targets were added. However, when downstream genes of the predicted genes in the same operons were included, some of the measurements become significant (*i.e*. characteristic path length, density in the PPI network and density in the transcription regulatory network). This may indicate that certain properties may still surface when there is a large enough sample size. This may improve as the number of available experimentally validated targets increases and as the available prediction methods are optimized.

## Authors' contributions

THW prepared the sRNA dataset, TargetRNA prediction and the centrality calculations. IYC handled the IntaRNA target prediction. LJC assisted in the interpretation of data. HCH and WVN provided direction and guidance. All authors read and approved the final manuscript.

## Competing interests

The authors declare that they have no competing interests.

## Supplementary Material

Additional file 1 **Complete centrality measurements for the RNA targets in the cellular networks**. Measurements for prediction-only results and predictions along with their downstream targets are included. Z values are reported for all of the analysis. Those with significant values (*p *< 0.05) are indicated in boldface and those close to a *p *value of 0.05 are underlined. Click here for file

Additional file 2 **Robustness of the experimental results**. (A) Measurements did not drastically change upon random removal and addition of protein-protein interactions. (B) Similar plot for robustness of the experimental results in transcription regulatory network. Click here for file

Additional file 3 **The gadY sRNA-targets in the transcription regulatory network**. *GadY *target *gadX *shows ranks that are significant for in-degree (9), out-degree (20), betweenness (0.00112), and closeness (0.398). *GadX *controls the transcription of pH-inducible genes and regulates acid resistance. The dark green lines represent experimentally verified regulation and the dark yellow lines represent predicted regulation. The teal and yellow lines indicate indirect regulation of genes downstream (in the operon) of experimentally verified or predicted direct targets, respectively (e.g., gadC is located downstream of gadB in the same operon). Nodes with pink borders represent transcription factors. Dashed lines indicate genes in the same operon, with the direction pointing from relative upstream to relative downstream. The operon relationships are only shown for concerned sRNA targets. Arrow, T, and diamond heads represent positive, negative, and dual regulators, respectively. Circular heads represent predicted, thus unknown, regulation. There are also several other predicted targets, but they are not present in the TR network. Click here for file
