# Supplementary material for: Modularity of Escherichia coli sRNA regulation revealed by sRNA-target and protein network analysis
Source: BMC Bioinformatics. 2010 Oct 15;11(Suppl 7):S11. doi: 10.1186/1471-2105-11-S7-S11 (PMC2957679; doi:10.1186/1471-2105-11-S7-S11)

Additional file 2: Robustness of the experimental results

(A) Measurements did not drastically change upon random removal and addition of protein-protein interactions. (B) Similar plot for robustness of the experimental results in transcription regulatory network.

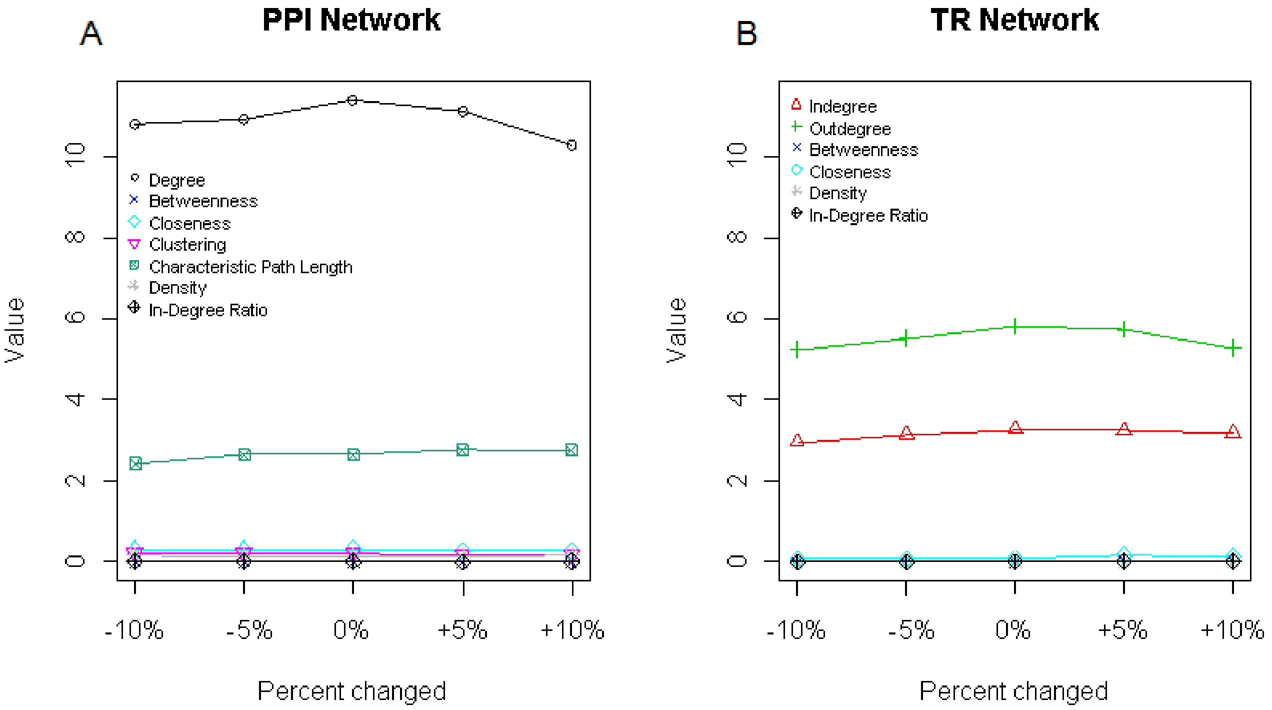

Supplement: Additional file 2 — Robustness of the experimental results. (A) Measurements did not drastically change upon random removal and addition of protein-protein interactions. (B) Similar plot for robustness of the experimental results in transcription regulatory network. [file 1471-2105-11-S7-S11-S2.pdf]
