# Supplementary material for: Modularity of Escherichia coli sRNA regulation revealed by sRNA-target and protein network analysis
Source: BMC Bioinformatics. 2010 Oct 15;11(Suppl 7):S11. doi: 10.1186/1471-2105-11-S7-S11 (PMC2957679; doi:10.1186/1471-2105-11-S7-S11)
Supplement: Additional file 3 — The gadY sRNA-targets in the transcription regulatory network. GadY target gadX shows ranks that are significant for in-degree (9), out-degree (20), betweenness (0.00112), and closeness (0.398). GadX controls the transcription of pH-inducible genes and regulates acid resistance. The dark green lines represent experimentally verified regulation and the dark yellow lines represent predicted regulation. The teal and yellow lines indicate indirect regulation of genes downstream (in the operon) of experimentally verified or predicted direct targets, respectively (e.g., gadC is located downstream of gadB in the same operon). Nodes with pink borders represent transcription factors. Dashed lines indicate genes in the same operon, with the direction pointing from relative upstream to relative downstream. The operon relationships are only shown for concerned sRNA targets. Arrow, T, and diamond heads represent positive, negative, and dual regulators, respectively. Circular heads represent predicted, thus unknown, regulation. There are also several other predicted targets, but they are not present in the TR network. [file 1471-2105-11-S7-S11-S3.pdf]

Additional file 3: The gadY sRNA-targets in the transcription regulatory network

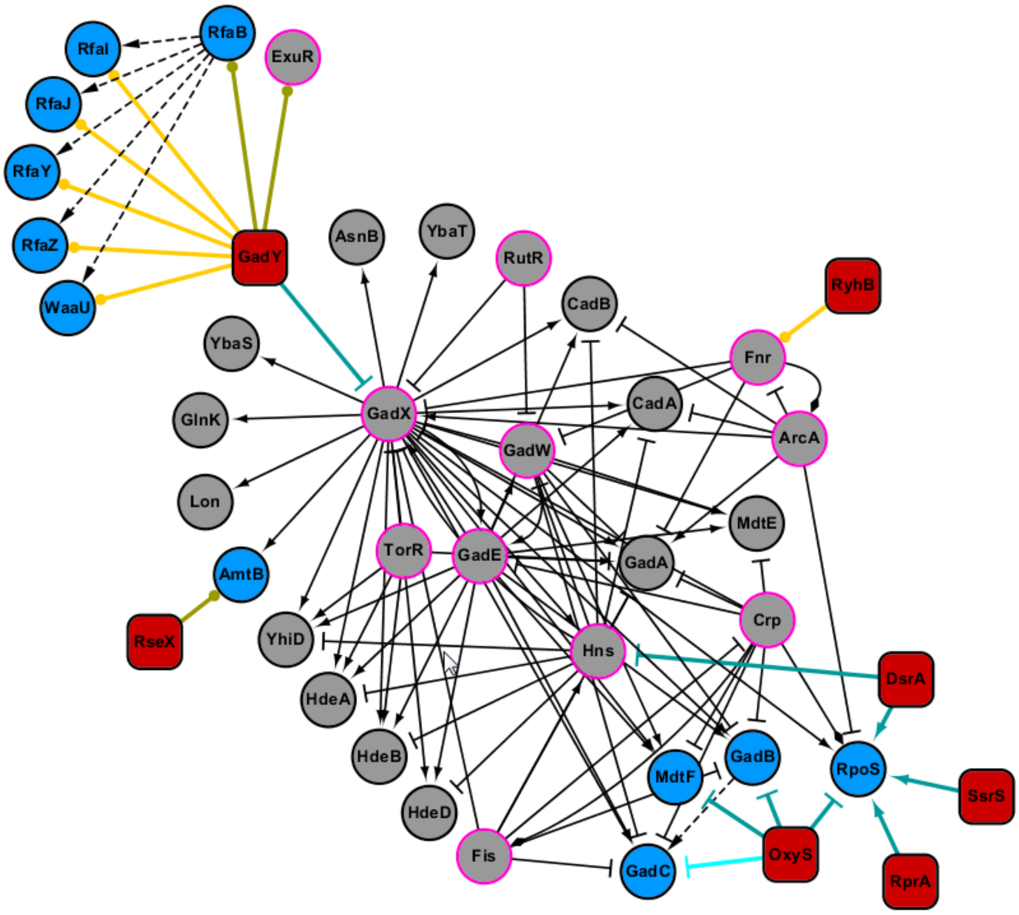

GadY target gadX shows ranks that are significant for in-degree (9), out-degree (20), betweenness (0.00112), and closeness (0.398). GadX controls the transcription of pH-inducible genes and regulates acid resistance. The dark green lines represent experimentally verified regulation and the dark yellow lines represent predicted regulation. The teal and yellow lines indicate indirect regulation of genes downstream (in the operon) of experimentally verified or predicted direct targets, respectively (e.g., gadC is located downstream of gadB in the same operon). Nodes with pink borders represent transcription factors. Dashed lines indicate genes in the same operon, with the direction pointing from relative upstream to relative downstream. The operon relationships are only shown for concerned sRNA targets. Arrow, T, and diamond heads represent positive, negative, and dual regulators, respectively. Circular heads represent predicted, thus unknown, regulation. There are also several other predicted targets, but they are not present in the TR network.
